# Chilaiditi Syndrome: A Rare Case and Clinical Insights for Diagnosis and Management

**DOI:** 10.7759/cureus.48932

**Published:** 2023-11-16

**Authors:** Frederico S Silva, Joana Moutinho, Tiago Vasconcelos, Inês G Simões

**Affiliations:** 1 Internal Medicine, Centro Hospitalar Universitário do Algarve - Unidade de Portimão, Portimão, PRT

**Keywords:** hepato-diaphragmatic interposition, diagnostic challenges, clinical awareness, radiological significance, chilaiditi syndrome

## Abstract

Chilaiditi syndrome is a rare medical condition characterized by the interposition of a hollow organ, usually the colon, between the liver and diaphragm, leading to abdominal pain, discomfort, bloating, constipation, or nausea; in more severe instances, respiratory symptoms may manifest due to pressure on the diaphragm. The exact cause remains unclear but is thought to present along with various factors such as anatomical anomalies (hepato-diaphragmatic interposition and intestinal malrotation) and chronic conditions (cirrhosis or chronic obstructive pulmonary disease). This case report presents a 78-year-old male with rapid deterioration, confusion, and mild abdominal discomfort. Clinical and radiological examinations confirmed Chilaiditi syndrome, highlighting the challenges in diagnosis. Management strategies range from conservative approaches to surgical interventions, emphasizing the need for increased clinical awareness among physicians to ensure accurate and timely interventions. This case report underscores the importance of recognizing this rare condition.

## Introduction

Chilaiditi syndrome is an uncommon medical condition allied with a peculiar radiographic image, where a hollow organ becomes interposed between the liver and diaphragm, causing digestive issues, mainly abdominal pain, and potentially cardiorespiratory problems. Chilaiditi's sign is identified when these specific radiological features are observed in an asymptomatic patient [1]. The exact cause remains unknown, although it could be linked to various factors such as anatomical anomalies, chronic constipation, aerophagia, cirrhosis, or chronic obstructive pulmonary disease [2].

## Case presentation

We report the case of a 78-year-old male who arrived at the emergency department experiencing a sudden decline in health, characterized by rapid onset of shortness of breath, confusion, and vague abdominal discomfort accompanied by mild constipation. The patient was able to confirm the absence of chest pain, vomiting, fever, chills, night sweats, black tarry stools, or diarrhea. He had a medical history notable for high blood pressure and hypertensive heart disease with heart failure with preserved left ventricle ejection fraction. The patient reported no history of diabetes mellitus, ischemic heart disease, or prior surgeries. He also denied any use of tobacco, alcohol, or illicit drugs, and his family history showed no significant health issues.

Upon arrival at the emergency department, the patient exhibited normal vital signs and respiratory functions. Although mildly distressed and confused during the examination, he remained oriented to person and place. Clear lung sounds were noted, with rhonchi in the right hemithorax. Cardiovascular assessment revealed a blood pressure of 134/70 mmHg and a regular heart rate of 94 beats per minute. Abdominal examination showed no abnormalities, tenderness, or signs of inflammation.

The most relevant laboratory analysis (Table 1) was consistent with normocytic and normochromic anemia, leukocytosis with neutrophilia, and elevated C-reactive protein. Arterial blood gas on room air showed compensated metabolic acidosis with hypoxemia.

**Table 1:** Relevant laboratory bloodwork findings on admission. INR, international normalized ratio; APTT, activated partial thromboplastin time; Na+, sodium; Cl-, chloride; K+, potassium; AST, aspartate transferase; ALT, alanine transaminase; GGT, gamma-glutamyl transferase; ALP, alkaline phosphatase; LDH, lactate dehydrogenase; PaCO_2_, partial pressure of carbon dioxide; PaO_2_, partial pressure of oxygen; HCO_3_, bicarbonate; SaO_2_, oxygen saturation

Variable	Patient results	Normal values
Hemoglobin (g/dL)	11.9	13.8-17.2
Leucocytes (×10^9^/L)	13560	5000-10000
Neutrophils (×10^9^/L)	9400	2500-8000
Platelets (×10^9^/L)	312	150-400
INR	0.9	<1.1
APTT (seconds)	32.6	30-40
C-reactive protein (mg/L)	104	<10
Total proteins (g/dL)	6.7	6.0-8.3
Albumin (g/dL)	4.6	3.4-5.4
Urea (mg/dL)	30	5-32
Creatine (mg/dL)	1.2	0.7-1.3
Na+ (mmol/L)	143	135-145
K+ (mmol/L)	4.2	3.6-5.2
Cl- (mmol/L)	103	96-106
AST (U/L)	23	8-33
ALT (U/L)	14	7-56
GGT (U/L)	19	0-30
ALP (U/L)	80	44-147
LDH (U/L)	240	105-233
pH	7.4	7.35-7.45
PaCO_2_ (mmHg)	36	35-45
PaO_2_ (mmHg)	58	75-100
HCO_3_ (mmol/L)	20	22-26
Lactate (mmol/L)	0.9	<2.0
SaO_2_ (%)	89	95-100

**Figure 1:**
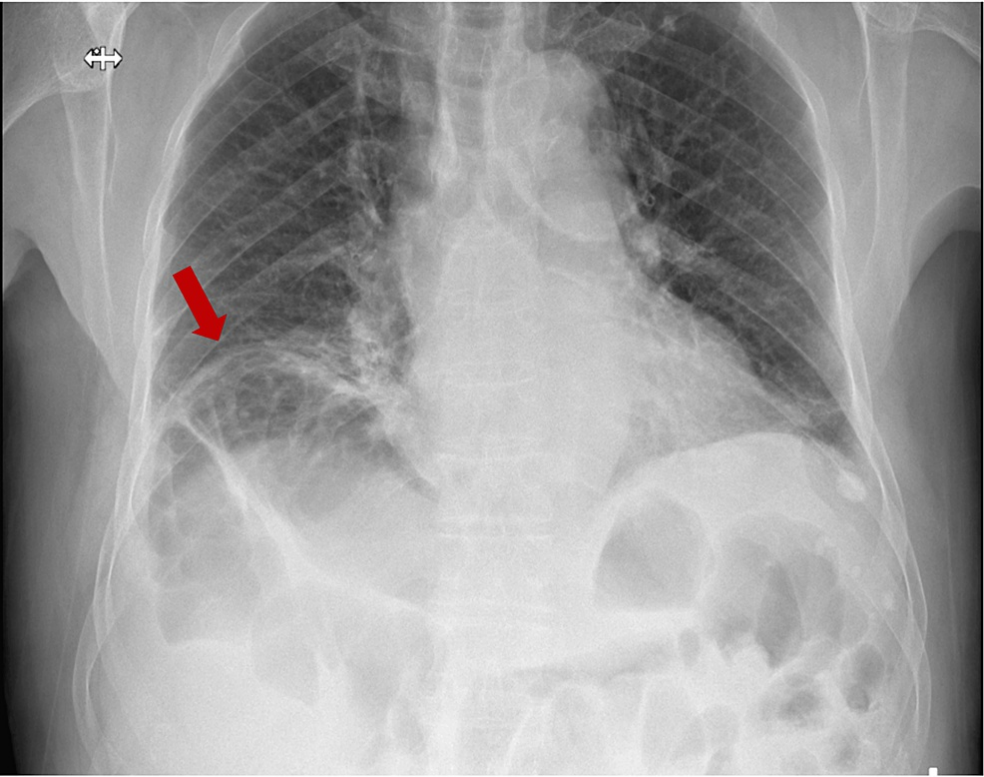
Thoracic X-ray with the red arrow indicating the raised hemicupola and colonic ansae beneath the diaphragm.

An anterior-posterior chest radiography was conducted to rule out other potential causes of the symptoms mentioned. The X-ray showed the presence of air beneath the right diaphragmatic cupola (Figure 1).

**Figure 2:**
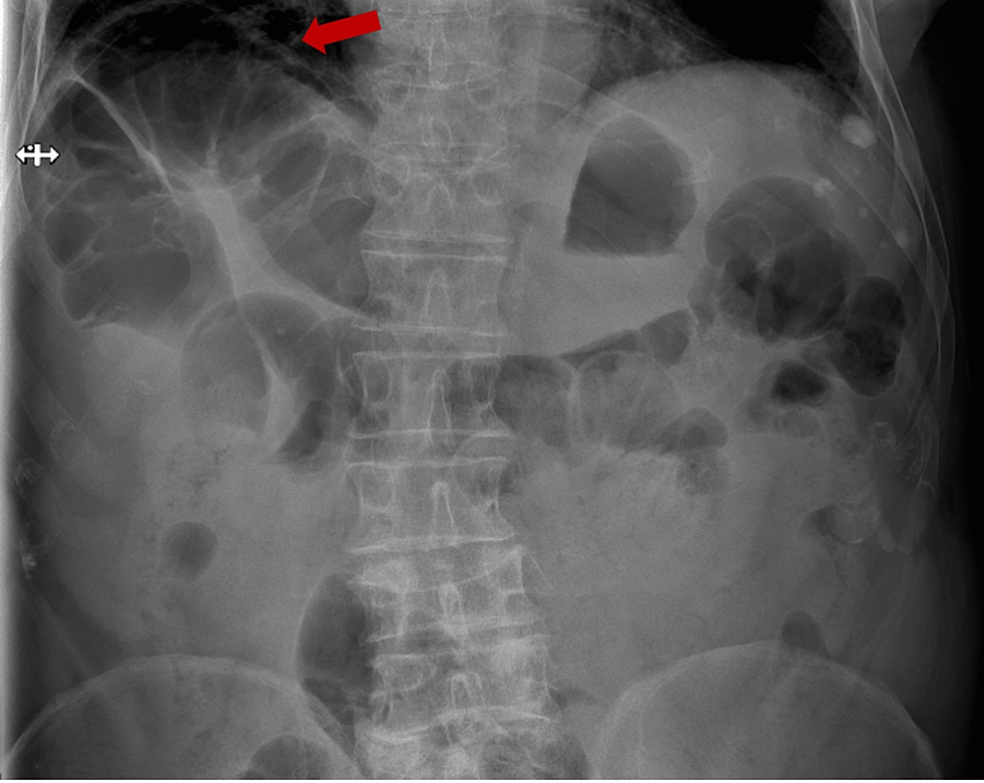
Abdominal X-ray displaying the anterior interposition of bowel loops in relation to the liver, indicated by the red arrow.

Comparison with previous X-rays confirmed the existing condition of hepatic diaphragmatic interposition of the colon loop, even though this finding had not been previously described or extensively documented. An X-ray of the abdomen was performed to obtain a clearer view of the issue (Figure 2).

**Figure 3:**
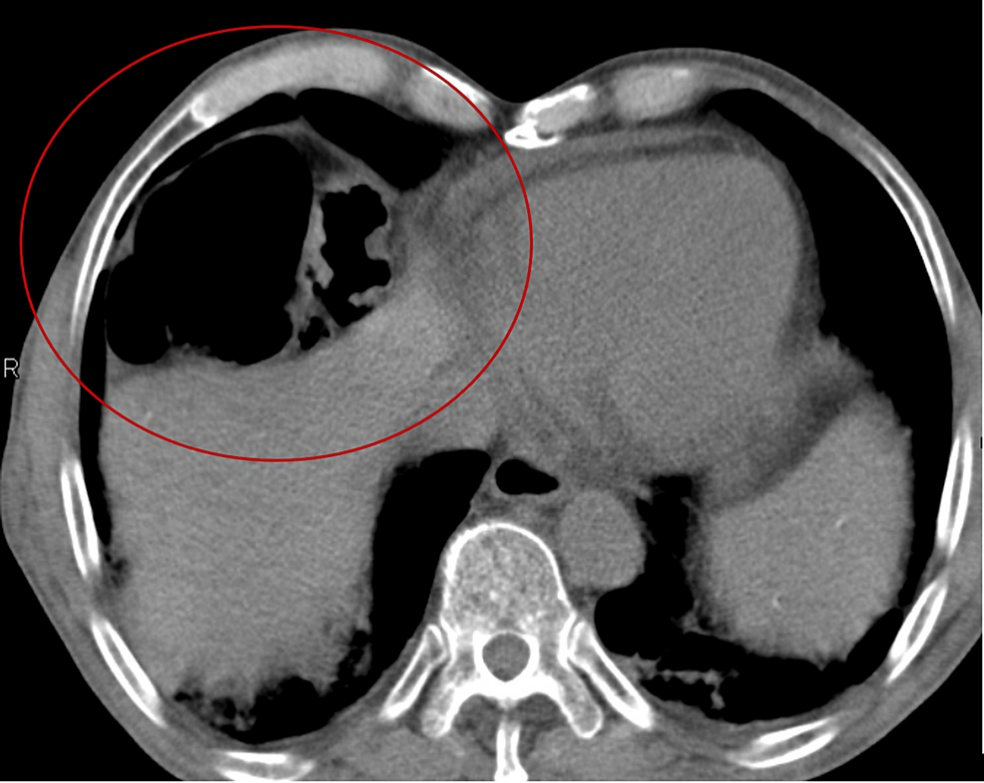
Abdominal CT scan revealing a colon loop between the diaphragm and the liver, as indicated by the red delineation. CT: computed tomography

The patient was admitted to the hospital, and empirical antibiotic treatment was started for a community-acquired respiratory infection. While hospitalized, an abdominal computed tomography (CT) scan was conducted to further assess the radiological signs suggesting Chilaiditi syndrome. The images showed that the observed air pocket under the raised right diaphragmatic copula was, in fact, a loop of the colon (Figure 3).

There were no signs of free air or fluid. From a radiological standpoint, this condition was identified as Chilaiditi's sign. Yet, with the patient exhibiting symptoms attributed to Chilaiditi syndrome, this diagnosis was included in the list of issues to be addressed during the hospital care.

Since the image results and clinical assessment indicated no complications, a conservative approach was chosen. The patient exhibited gradual improvement in respiratory and abdominal symptoms. Subsequently, the patient was discharged and continued to do well during follow-up appointments.

## Discussion

Described by Dr. Demetrius Chilaiditi in 1910, the radiological phenomenon of air accumulation under the diaphragm, resulting from the colon positioning itself between the right hemidiaphragm and the liver, was coined as Chilaiditi's sign [3]. More than a century later, this phenomenon remains noteworthy due to its simplicity of detection through plain chest radiography and its significant clinical relevance.

It occurs more frequently in males (with a ratio of 4:1) and is typically an unexpected discovery, appearing in approximately 0.02%-0.14% of radiological studies conducted for various reasons [4].

The specific cause for this condition remains uncertain, but it is suspected to involve the abnormal positioning of the colon in the space between the liver and the diaphragm. Potential contributing factors include ligamentous laxity and the elevation of the right diaphragmatic copula due to phrenic nerve paralysis, liver cirrhosis, or chronic obstructive pulmonary disease. Usually, the diagnosis is made incidentally during imaging tests conducted for unrelated diagnostic reasons. Abnormal colon positioning is frequently identified in plain X-ray images, sometimes displaying colonic air that resembles air beneath the diaphragm. While chest and abdominal X-rays are less sensitive for diagnosis, CT scans offer more accurate and reliable results [5].

Symptoms can vary widely, ranging from less urgent issues such as constipation, loss of appetite, and vomiting to urgent medical emergencies such as chest pain, difficulty breathing, abdominal pain, volvulus, and bowel obstruction. Patients may present with diverse symptoms, but abdominal pain, ranging from chronic intermittent discomfort to sudden and severe pain, is a common element in most cases. In our patient's case, symptoms related to Chilaiditi syndrome were somewhat overshadowed by the clinical findings of a respiratory tract infection. However, these symptoms were not dismissed, and recognizing Chilaiditi syndrome was crucial as it alerted us to the potential complications associated with this condition [5].

In most instances, including this patient's situation, conservative treatment is adequate. This approach typically includes rest, intravenous fluids, and relieving bowel pressure to alleviate symptoms. However, in cases involving intricate abdominal problems such as obstruction, volvulus, or perforation, surgical intervention most likely becomes necessary [6].

## Conclusions

The rarity of Chilaiditi syndrome renders it susceptible to misdiagnosis. This case underscores the critical significance of heightened clinical awareness concerning this syndrome among physicians. Such awareness is pivotal in minimizing the incidence of unwarranted surgical interventions, thereby ensuring appropriate and accurate medical management.
